# Structured and sustained family planning support facilitates effective use of postpartum contraception amongst women living with HIV in South Western Uganda: A randomized controlled trial

**DOI:** 10.7189/jogh.11.04034

**Published:** 2021-06-05

**Authors:** Esther C Atukunda, Godfrey R Mugyenyi, Angella Musiimenta, Angela Kaida, Elly B Atuhumuza, Edward J Lukyamuzi, Amon G Agaba, Celestino Obua, Lynn T Matthews

**Affiliations:** 1 Mbarara University of Science and Technology, Mbarara, Uganda; 2 Simon Fraser University, Faculty of Health Sciences, Burnaby, Vancouver, Canada; 3 University of Alabama at Birmingham, School of Medicine, Division of Infectious Diseases, Birmingham, Alabama, USA

## Abstract

**Background:**

Despite low pregnancy intentions, many women accessing contraception discontinue use, increasing the risk of unwanted pregnancies among women living with HIV (WLWH). We evaluate whether a family planning support intervention, inclusive of structured immediate one-on-one postpartum counseling, and a follow-up mechanism through additional health information and SMS reminders affects continuous contraceptive use and pregnancy incidence among recently postpartum WLWH.

**Methods:**

We performed a randomized controlled trial between October 2016 and June 2018 at a referral hospital in southwestern Uganda. We included adult WLWH randomized and enrolled in a 1:1 ratio to receive family planning support or standard of care (control) and completed an interviewer-administered questionnaire at enrolment, 6 and 12 months postpartum. Our two primary outcomes of interest were; continuous use of contraception, and incidence of pregnancy. Secondary outcomes included contraception uptake, method change, discontinuation and pregnancy intentions. The trial was registered with clinicaltrials.gov (NCT02964169).

**Results:**

A total of 317(99%) completed all study procedures. Mean age was 29.6 (SD = 6.0) vs 30.0 (SD = 5.9) years for the intervention vs control groups respectively. All women were enrolled on ART. Total women using contraception continuously were 126 (79.8%) in the intervention compared to 110 (69.2%) in control group (odds ratio (OR) = 1.75; confidence interval (CI) = 1.24-2.75, *P* = 0.003). Pregnancy rates were 2% (N = 3) in the intervention vs 9% (N = 14) in the control group (OR = 0.20, 95% CI = 0.05-0.62, *P* = 0.006). Pregnancy intention was lower in the intervention vs control group (OR = 0.23, 95% CI = 0.08-0.64, *P* = 0.002). Women actively enrolled on contraception reduced more in the control compared to the intervention group (OR = 3.92, 95% CI = 1.66-9.77, *P* = 0.001). Women enrolled on each contraceptive method did not differ by group except for implants. More women initiating contraception use within three months postpartum had better continued use for either intervention (N = 123, 97.6% vs N = 3,2.4%) or control group (N = 86,78.2% vs N = 24,21.8%). Method-related side effects were less reported in the intervention group (OR = 0.25, 95% CI = 0.10-0.60, *P* = 0.001).

**Conclusion:**

We found that sustained and structured family planning support facilitates continuous use of contraception and lowers rates of pregnancy amongst postpartum WLWH in rural southwestern Uganda. Women who initiated contraception within three months postpartum were more likely to maintain continuous use of contraception than those initiating later. Further evaluation of actual and perceived facilitators to the continuous contraception use by this support intervention will help replication in similar settings.

**Trial registration:**

NCT02964169

Supporting women living with HIV (WLWH) to delay or prevent unwanted pregnancy may improve women’s health [1]. While many WLWH want to have children [2,3], in a recent survey of immediately post-partum WLWH in southwestern Uganda, over 95% wanted to avoid unwanted pregnancies in the next 12 months [4]. However, despite low pregnancy intentions, the overall postpartum contraceptive prevalence for WLWH in Uganda remains at 57%, with up to 50% of pregnancies reported as unplanned [5]. Many women accessing contraception therefore, may inconsistently use or discontinue use without switching to another alternative and effective method, which can lead to unwanted pregnancies, perinatal HIV transmission, and other pregnancy complications [6,7]. According to some studies, about 15% of breastfeeding women conceive before resumption of menses, 28% women conceive while exclusively or almost exclusively breastfeeding [8,9]. These risks and complications are higher among WLWH [2,3]. Correct use of family planning methods can avert 10% of child mortality and more than 30% of maternal deaths by promoting spacing of pregnancies [7,10,11]. Pregnancies in the postpartum period pose the greatest risk for the health of women and their infants, with increased risks of adverse health outcomes [12].

Using a birth control method soon after childbirth is very crucial in ensuring unintended pregnancies are avoided in more than 95% of postpartum women who want to avoid pregnancy in the next 24 months [12]. Data suggest that women who began contraception before the return of their menses were more likely to continue contraception use by the end of their first year postpartum compared to those who initiated a family planning method after the return of their menses [11]. Other scholars have noted up to 40 percent of women with an unintended pregnancy while accessing contraception in Missouri, USA were using the method incorrectly or inconsistently [13]. This rate may be higher in sub-Saharan Africa and Uganda. Some behavioral change interventions targeting to improve uptake, continuation, and proper use of postpartum family planning in similar low resource settings include; easing access to family planning services, increasing supply and method choice, enabling women to immediately switch to preferred or more acceptable and effective methods when they encounter problems, and improving follow-up mechanisms (eg, appointment or refill reminders via mobile technology) [6,14]. However, postpartum contraceptive uptake remains low [12] despite many health facility encounters women and or couples have around the postpartum period. These encounters would potentially be a cost-effective, convenient, efficient and reliable “touch point” to initiate and or provide a woman with an effective family planning method. Limited research has been conducted to understand contraception discontinuation and unintended pregnancies during the early postpartum period, especially among postpartum WLWH whose probability of contraceptive failure has been reported to rise sharply over time [15,16].

We performed a randomized controlled trial to evaluate whether a family planning support intervention voucher, inclusive of structured immediate postpartum counseling, and a follow-up mechanism providing additional health information and SMS reminders has a measurable impact on continuous use of contraception, and pregnancy incidence at 12 months postpartum among recently postpartum WLWH delivering at a publically-funded regional referral hospital in rural, southwestern Uganda. We hypothesized that improved family planning support would improve continuous use of effective FP by reducing delayed refills, discontinuation, improving rates of switching to an alternative and effective contraception method, and thus improve protection against unintended pregnancies within the first year following childbirth. Earlier analyses showed that very few women in this cohort (N = 9, 2.8%) wanted to have another child within the first year following childbirth, with women in the intervention group more likely to initiate contraception within 8 weeks postpartum [4,17]. In this analysis we present the 12-month data on continuous contraceptive use and pregnancy incidence.

## METHODS

### Study design and setting

This analysis includes 12-month exit data collected from WLWH enrolling into a randomized controlled trial at the maternity ward of a regional referral hospital in southwestern Uganda. A complete description of the study’s six-month follow-up period has been published [4]. This interim analysis presented the initiation of a modern family planning method within 8 weeks postpartum as a primary outcome of interest. Mbarara Regional Referral Hospital (MRRH) is a publically-funded teaching hospital serving 10 districts, with a population of over 5 million people. The study aimed to evaluate the effect of family planning support vs standard of care on contraceptive use at 12 months postpartum (NCT02964169). The hospital is equipped with trained staff, midwives, and obstetricians able to offer comprehensive family planning services. Women accessing care at this hospital represent varied social and demographic backgrounds. The hospital performs over 12 000 deliveries annually and reports a 13% HIV prevalence among women (hospital records). Due to structural and capacity challenges at the referent hospital site, routine discharge is often completed without family planning counseling.

### Participants and recruitment

This study was initiated in October 2016 and enrolment ended in May 2017. Eligible participants were WLWH 18 years of age or older, having a male sexual partner and/or anticipating one within the next 2 years, admission into a postnatal ward regardless of pregnancy outcome and qualified for any family planning methods available. The exclusion criteria included: 1) HIV-negative, 2) history of hypersensitivity to latex, 3) no male sexual partner and/or not anticipating one for the next 2 years, 4) only sexual partner has had vasectomy, 5) resides and works more than 20km from the study site, or 6) inability to complete informed consent process as assessed by the study nurses. A primary partner was defined either as a regular spouse, who is also a regular sexual partner, or the most recent sexual partner if no main partner was named. Trained research assistants (RAs) approached WLWH in the postnatal ward within 12 hours after delivery to capture all women delivering at this facility. RAs obtained voluntary written informed consent from all eligible participants in the local language in a private area of the hospital. All consenting participants gave written informed consent, or for those who could not write, a thumbprint was made on the consent form.

### Intervention design

#### Family planning voucher intervention

Following delivery, the women randomized to the intervention group were counseled and given a family planning voucher by the study nurse. Structured, immediate postpartum counseling was offered in a clinic setting, in a private room by a well-trained study nurse and lasted up to 40 minutes as previously reported [4]. The one-on-one educational counseling provided face-to-face standardized (a list of items to talk about was generated) information to the woman and primary sexual partner (if available) on the available contraceptive methods, family size choices and desires, medical eligibility for the different contraceptive methods, dual contraception, when to start contraception, how to use methods, potential side effects and benefits/effectiveness, and where to access family planning methods. Women were given opportunity to ask questions to facilitate women’s informed choice to any of the five freely-available family planning methods at MRRH (including condoms, injectables, contraceptive pills, copper IUDs, and contraceptive implant). WLWH were also counseled on standard days method (SDM), and lactational amhenorrea method (LAM). The same voucher and counseling was also given to the male sexual partner, when available, due to its identified effect on family planning utilization [18]. The voucher was used as an incentive to motivate women to seek/demand and access family planning easily at family planning clinics. A well-trained nurse was available at the postnatal clinic to identify women with vouchers to access the relevant service provider within one hour of arrival.

Although family planning is freely-available in public health facilities, stock outs, especially of the long-acting contraceptive methods (implants and IUDs), attributed mainly to supply chain challenges are common [19]. The study promoted minimal stock outs of all methods at MRRH during the study period through regular involvement in forecasting and ordering. Private facilities rarely experience contraceptive methods stock outs [19], which are fairly affordable, and thus the family planning voucher also offered an opportunity for free administration (eg, injection, IUD placement, implant) of a contraceptive method purchased (by participants) outside of the public health care facility. For this study, women who reside and or work within 20km from MRRH were enrolled, thus all women were in close-proximity to a facility with family planning services. The voucher was offered for free, had an expiration of 3 months from the date of delivery and included detailed information about side effects for the different contraceptive methods as well as a general overview on benefits of family planning [4]. Within 3 months postpartum, the women were expected to have returned to a health facility for at least 2 of their scheduled routine postnatal visits and or immunization appointments.

**Figure 1:**
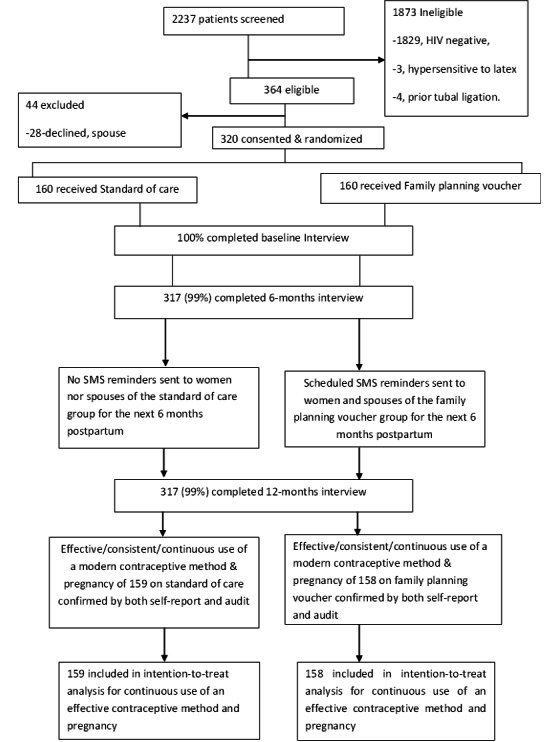
Trial profile.

After 6 months postpartum, the women who selected oral contraceptive pills were sent daily scheduled SMS reminders [adherence support] for the first 4 months, then weekly reminders for the next 2 months (**Figure 1**). This level of SMS support has been found to have a positive impact on adherence to ART [20]. Enrolled sexual partners/regular spouses of women in intervention arm also received these reminders weekly [but not daily or monthly]. The scheduled/timed reminders were also sent monthly if one chose an injectable contraception. Daily reminders were sent to women who chose male or female condoms. Routine reviews on family planning were done for all women alongside their routine visits at the HIV clinic or post-natal PMTCT visits.

#### Control group

In order to align the control group with guidelines-based standard of care, women in the control group were offered routine family planning counseling at discharge as defined by the Uganda clinical guidelines [21] by a well-trained ward nurse and documented in the Ministry of Health discharge form. The control group was not given a voucher nor received any SMS reminders.

Women from both groups were invited to start any available family planning method of their choice prior to discharge. The choice and place of family planning was entirely up to the participants regardless of group. All women accessing services at the hospital family planning clinic received care by a trained nurse to counsel and administer a chosen family planning method, beside the study nurse, who doubled as a dedicated contact person at the same clinic. Women were followed for one year. All participants completed interviewer-administered interviews at baseline, six and twelve months postpartum. Interviews were conducted by two trained research assistants fluent in English and the main local language in a private office space or any other private space of their choice. Each interview took about 30-45 minutes. Permission to contact spouses/sexual partners was obtained from all enrolled women. If permission-to-contact was given, a spouse/ sexual partner was contacted, enrolled and interviewed at baseline, 6 and 12 months for both groups. The spouses for controls were not given vouchers nor sent SMS reminders.

### Randomization and blinding

A study biostatistician generated a randomization list with a block size of 20, totaling 160 participants equally in each of the two groups into which mothers could be randomly assigned and enrolled. The aim of the study and details of the procedures to be involved in the trial, were explained before randomization. Once mothers consented to participate in the study, a study number was allocated by the research assistant by taking the next in a series of similar opaque envelopes provided to conceal allocation of groups. These opaque envelopes were labeled with computer-generated list of numbers with group allocation. The intervention provider and study participants were not blinded to the intervention, as the study design and nature of the intervention did not allow it. However, the research assistants were blinded to the group allocation until eligibility and study participation was confirmed. They were also blinded to the hypothesis of the study.

WLWH were screened for eligibility and enrolled equally into the intervention arm (Family planning support) and standard of care (control group) between October 2016 and May 2017. A different RA from the one enrolling participants was trained to specifically collect follow-up data to limit social desirability bias. Data was collected electronically. The data analyst was blinded to the group allocated to different study participants. A transport refund of US$3 was given on each visit.

### Study measures

A blood sample was drawn at baseline to confirm HIV status and measure CD4 cell count. A structured face-to-face questionnaire was completed at enrollment to collect information on socio-demographics, depression and health [22], reproductive history, partnership dynamics (eg, HIV serostatus disclosure, partner HIV-serostatus), perception, use and knowledge of contraception, decision making [23-28], food insecurity [29,30], alcohol use in the last one year [31], HIV stigma [32], social support [33], and pregnancy intentions or aspiration [34-36]. Experiences with adult physical and sexual violence by an intimate partner within the past 12 months was assessed using standardized questions [37].

### Study outcome

Our primary outcomes of interest for this trial were 1) effective contraception use, defined as consistent use (both self-report and observational chart review by a study nurse at the family planning clinic to identify and confirm consistent use) of a family planning method within 12 months postpartum, and 2) pregnancy incidence, defined as the rate of pregnancies in the next one year following enrolment. Missing family planning method for more than 2 calendar days in a cycle/mo for all contraceptive methods (except for more than a week for injectables) was defined as inconsistent use of contraception. Outcomes from both reports were evaluated to confirm the internal validity and consistency of the two measures. A family planning chart or card is usually provided at any facility providing family planning services, and routinely presented by the women and filled out by any attending HCP initiating, switching or renewing a contraceptive method. Blister packs for oral contraceptives were inspected by the study RA whenever available. Secondary outcomes included family planning uptake at 6 and 12 months postpartum, change of family planning method, pregnancy intentions, contraceptive discontinuation (abandonment or late refills of any contraceptive method for more than 1 week except more than 1 month for injectables) and reasons for discontinuation. Consistent use for condoms or diaphragm as an effective family planning method was defined as “use every time one had sex”. Although postpartum counseling on contraceptive methods focused on the five methods; condoms, injectables, contraceptive pills (including progestin-only pills for breastfeeding mothers), copper IUDs, and contraceptive implants as provided at MRRH, modern family planning was defined as use of these five and any other methods such as diaphragm, cervical cap which participants could have obtained from other facilities. Switching was defined as any change of a family planning method. Pregnancy tests were done at 6 and 12-month follow up visits. Follow-up tools also contained specific questions to explore and document a pregnancy occurrence within the last 6 months. The women were further instructed to inform/notify the study nurse upon any other pregnancy diagnoses during the study period.

### Sample size and statistical analysis

Provision of a family planning voucher has a significant impact on contraceptive uptake and long-term contraceptive use by an increase of 18 percentage points within 2 years of the reporting period among postpartum women [18]. The current contraceptive uptake among WLWH is 45% [2]. We therefore hypothesized that improved and focused family planning support through a voucher will increase effective contraceptive use among WLWH to at least 63% within a follow-up period of one year postpartum. Allowing for a two-sided type I error of 5%, we required 320 postpartum women (with equal numbers of participants in the intervention and control groups) to enable 90% power to demonstrate a significant difference in consistent (effective) contraceptive use (our first primary outcome) between groups.

For the second primary outcome, Nieves and colleagues [2] documented a pregnancy desire/ aspiration rate of 33% for sexually active WLWH attending ART clinic. Specifically, Snow and colleagues [1] also reported a lower and significant likelihood in desiring more children in future of 27.7% among married or cohabiting HIV-positive women when compared to 56.4% among HIV-uninfected women. Improved family planning accessibility and support offered through a voucher reduced the rate of births in the next year following the intervention to 6.8% [18]. We therefore hypothesized a decrease in pregnancy or births in the one year following the intervention to 6.8% with improved family planning availability and support among the recent post-partum WLWH. Allowing for a two-sided type I error of 5%, we required a total of 80 women in each of the two groups to have a 95% chance of detecting a significant difference in pregnancy rates (our second primary outcome) between groups.

We described demographic and clinical data for the cohort using standard descriptive statistics. The Household Food Insecurity Access Scale (HFIAS) was calculated as recommended [38]. We compared dichotomous outcomes between study groups by estimating crude odds ratios with 95% confidence intervals, and testing for differences between the two groups. We estimated *P*-values with χ2 testing using a level of significance of 0.05. We compared continuous outcomes and estimated *P*-values using studentized *t*-tests. All primary and secondary outcomes were analyzed using intention-to-treat analyses (although no participants were wrongly allocated a group [23]. Although our study was fully randomized, the differences in baseline characteristics noted between study groups was assessed for confounding by fitting multivariable logistic regression models. As per the revised CONSORT guidelines for reporting randomized trials [26], we assessed for sub-group effects for the following characteristics by testing the significance of interaction terms in a multivariable regression model: 1) children living in household below 18 years of age (dichotomized into <3 children and ≥3 children categories), 2) parity (dichotomized into 1-3, >3), 3) Prenatal visits (<3 and ≥3 visits), 4) Household income (≤150 000 and >150 000 Ugandan Shillings), 5) involvement in any domestic violence (Involvement, no involvement), 6) Religion (Catholic, protestant, others), 7) disclosure to spouse, and 8) duration on ART (<4, 4-8 and >8 years). These sub-groups were not pre-specified but identified at data analysis stage when comparing different baseline characteristics across the two arms. A Mantel-Haenszel test was also done to control for each of these variables. All statistical analyses were performed using STATA version 13.0 (Statacorp, College Station, Texas, USA).

### Compliance with ethical standards

All human subjects’ ethical approvals were obtained from Institutional Review Committees of Mbarara University of Science and Technology (No.10/08-16) and Uganda National Council of Science and Technology, and registered with clinicaltrials.gov (NCT02964169). A research assistant trained in human participant research conducted informed consent procedures with eligible participants in the local language in a private area. A written informed consent was obtained from all eligible participants.

## RESULTS

Of the 2237 women screened for eligibility between October 2016 and May 2017, 364 participants were eligible (**Figure 1**). A total of 28 women declined participation because they had not yet disclosed their HIV sero-status to partner, and 16 declined due to the time commitment. Of the 1873 women excluded, 1829 were due to HIV-negative sero-status, residing and working outside catchment area (21), age below 18 years (16), history of tubal ligation (4), or reported history of hypersensitivity to latex (3). A total of 320 postpartum WLWH were randomized and enrolled equally into the family planning voucher and control arms of the study following delivery at MRRH. Three hundred and seventeen (99%) of enrolled participants completed all study procedures.

The characteristics of the enrolled women are presented in detail elsewhere [17] and summarized in **Table 1**. In brief, the mean age of participants was 29.6 (Standard Deviation [SD] = 6.0) and 30.0 (SD = 5.9) years for the control and family planning voucher groups, respectively. Mean CD4 count was 396 cells/mm^3^ (SD = 61) for those enrolled in control vs 393 cells/mm^3^ (SD = 64) in family planning voucher. At enrolment, half of the women in both the voucher (N = 87, 55%) and control (N = 86, 54%) groups wanted to have a child in 2 years postpartum. Over 80% of referent pregnancies in the voucher (N = 136, 86%) and control (N = 128, 81%) groups were reported as planned. All women were enrolled on ART, with mean ART duration of 5.1 (SD = 4.5) for those in the voucher group and 4.1 years (SD = 3.3) for those enrolled in the control condition. Almost half of participants (46%) attained education greater than primary (50% vs 43%). A small number of male sexual partners participated in the study including 18 (11%) and 21 (13%) for the voucher and control, respectively. Most of the women (N = 107, 70% vs N = 103, 69%) reported prior use of modern family planning methods. None of the women opted to start or receive immediate postpartum family planning before discharge. Other demographic and clinical characteristics were similar between the two groups as presented in **Table 1**.

**Table 1:** Baseline demographic and clinical characteristics of recently postpartum women living with HIV in Uganda, N = 317

Characteristics	Standard care (n = 159)	FP voucher (n = 158)
	**Mean (SD) or n (%)**	
Mean age (years)	29.6 (6.0)	30.0 (5.9)
Partner age (years)	34.4 (7.2)	34.9 (7.3)
Mean CD4 (SD)	396 (61)	393 (64)
Partner on ART	60 (48.4)	54 (42.9)
Mean duration on ART (years)	4.1 (3.3)	5.1 (4.5)
Duration on ART (years):		
<4	69 (54.3)	63 (50.4)
4-8	40 (31.5)	39 (31.2)
>8	18 (13.2)	23 (18.4)
Educational attainment greater than primary	68 (42.8)	79 (50.0)
Sexual partners contacted & enrolled in study	21 (13.2)	18 (11.4)
Religion:		
Catholic	41 (26.1)	25 (16.1)
Protestant	90 (56.6)	100 (63.3)
Others	26 (16.6)	30 (19.4)
Household children <18 years:		
0-1	45 (28.3)	45 (28.5)
2-3	92 (58.9)	75 (47.5)
≥4	22 (13.8)	38 (24.1)
Ever used any modern contraception in last 10 years	107 (69.5)	103 (68.7)
Used modern contraception in 2 years pre-pregnancy	61 (37.7)	60 (38.0)
Desire for pregnancy in 2 years	87 (54.7)	86 (54.4)
Desire for pregnancy in within 1 years	5 (3.5)	4 (2.6)
Partner desires another child in 2 years	94 (59.1)	90 (57.0)
Most recent pregnancy planned	136 (85.5)	128 (81.0)
Parity:		
1	25 (15.7)	28 (17.2)
2-3	92 (57.9)	71 (44.9)
>3	42 (26.4)	59 (37.3)
Prenatal visits attended:		
0-1	6 (3.8)	4 (2.5)
2-4	117 (73.6)	125 (79.1)
>4	36 (22.6)	29 (18.4)
Severe food insecurity*	30 (18.9)	31 (19.6)
Depression score^†^	4.2 (2.8)	4.6 (3.8)
Consumed alcohol in last 1 year	108 (67.9)	106 (67.1)
Household income (Ush):		
<100 000	91 (57.2)	74 (46.8)
100 000-150 000	31 (19.5)	31 (19.6)
>150 000	37 (23.3)	53 (33.5)
Monogamous household	128 (80.5)	135 (85.4)
Vaginal mode of delivery for last pregnancy	125 (78.6)	126 (79.8)
Domestic violence in current relationship	18 (14.4)	30 (23.8)
Disclosed HIV sero-status to sexual partner	133 (83.6)	138 (87.3)
Knows/sure about sexual partners sero-status	112 (70.4)	110 (69.6)
Takes part in decision making	87 (54.7)	90 (57.0)

SD – standard deviation, FP – family planning, ART – antiretroviral therapy, Ush – Ugandan shillings

*HFIAS>8 means severe food insecurity.

†This score ranges from1-48, with 0 indicating no depression.

**Figure 2:**
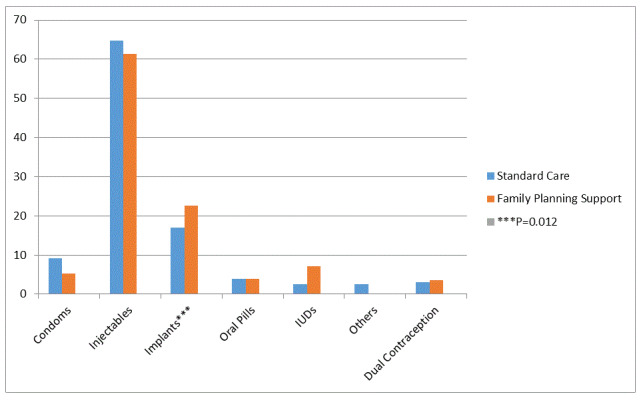
Percentage utilization of family planning methods at 12 months among those who initiated contraception (N = 308).

Both self-report and postnatal chart audit to evaluate contraceptive use generated identical outcomes, confirming the internal validity and consistency of the two measures. By 12 months postpartum (1st primary outcome), 126 (79.8%) women used an effective family planning method(s) consistently in the family planning support group compared to 110 (69.2%) women in control group (OR = 1.75; 95% CI = 1.24-2.75, *P* = 0.003, **Table 2**). Pregnancy rates (2nd primary outcome) were 2% (N = 3) in the first 12 months postpartum of the family planning intervention group compared to 9% (N = 14) in the control group (OR = 0.20; 95% CI = 0.05-0.62, *P* = 0.006). The desire/intention to have another child was lower in the intervention group compared to control group (OR = 0.23; 95% CI = 0.08-0.64, *P* = 0.002). The proportion of women who ever-enrolled on any family planning method in the last 12 months postpartum was not significantly different between groups (OR = 2.03, 95% CI = 0.50-8.28, *P* = 0.316). In this cohort, the proportion of women still actively enrolled on an effective family planning method at 12 months postpartum reduced for both groups, but more in the control compared to the intervention group (OR = 3.92; 95% CI = 1.66-9.77, *P* = 0.001). By 12 months postpartum, frequency of methods used differed by group for each family planning method (except for oral contraception): injectables were selected by most women (N = 194, 63%, *P* = 0.057, **Figure 2**) and 61% of this proportion was in the experimental arm vs 65% in the control arm. The proportion of women using implants followed at 19.8% (N = 61, 23% vs 17%, *P* = 0.012), with <10% of women in each group selecting condoms (5.5% vs 9.2%, *P* = 0.630), oral contraception (3.9% vs 3.9%, *P* = 0.933), and IUDs (7.1% vs 2.6% *P* = 0.091). About 3% of the women in the control arm and none in the intervention arm used standard days method, and or lactation amenorrhea method. Less than 4% used dual contraception in each of the groups. No women reported use of a diaphragm or cervical cap.

**Table 2:** Primary and secondary outcomes by treatment group

Outcomes	Routine care (n = 159)	FP voucher (n = 158)	Odds ratio (95% CI)	*P*-value
**Primary outcomes:**				
Confirmed continuous use of an effective contraceptive method at 12 months	110 (69.2%)	126 (79.8%)	1.75 (1.24-2.95)	0.003
Pregnancy in the 1^st^ year postpartum	14 (8.8%)	3 (1.9%)	0.20 (0.05-0.62)	0.006*
**Secondary outcomes:**				
Intention to be pregnant in the next 1 year postpartum	20 (12.6%)	5 (3.2%)	0.23 (0.08-0.64)	0.002
Ever been enrolled on any family planning method in last 1 year	153 (96.2%)	155 (98.1%)	2.03 (0.50-8.28)	0.316
Currently enrolled on family planning	123 (77.4%)	147 (93.0%)	3.92 (1.66-9.77)	0.001
Changed contraception in the last one year†	9 (5.7%)	12 (7.6%)	1.36 (0.54-3.22)	0.548
Continuous contraception use‡				
Initiated contraception at ≤3 months postpartum	86 (78.2%)	123 (97.6%)	2.98 (1.76-3.87)	<0.001
Initiated contraception at >3 months postpartum	24 (21.8%)	3 (2.4%)	0.11 (0.03-0.47)	<0.001
Discontinued family planning† for at least 1 week (2 weeks for injectables)	37 (23.3%)	15 (9.5%)	0.35 (0.17-0.65)	0.001
Reasons for discontinuation:§				
Wanting a child/death of a child	5 (3.1%)	3 (1.9%)	0.59 (0.11-1.88)	0.212
No longer needing protection	4 (2.5%)	5 (3.2%)	1.26 (0.22-3.18)	0.651
Method-related side effects	25 (15.7%)	7 (4.4%)	0.25 (0.13-0.58)	0.001
Spouse disapproval	8 (5.0%)	6 (3.8%)	0.75 (0.15-3.42)	0.613
Family planning inconvenient	2 (1.3%)	2 (1.3%)	1.01 (0.14-5.62)	0.916
Stock outs	0	0	-	-
Able to talk to others/spouse about FP	129 (81.1)	148 (98.7%)	3.442(1.97-6.15)	0.041

CI – confidence interval, FP – family planning

*Fisher exact test.

†Among N women in the control group and N women in the intervention group who ever initiated FP.

‡Only those reporting continued contraception use.

§Includes multiple responses of all N women exited within each of the study arms.

More women who started early use of contraception within 3 months postpartum had better continued use of contraception than those starting later than three months postpartum for either intervention (N = 123,97.6% vs N = 3, 2.4%) or control group (N = 86, 78.2% vs N = 24, 21.8%). Although no statistically significant differences were detected, more women in the intervention group changed contraceptive method within the course of one year postpartum (OR = 1.36; 95% CI = 0.54-3.22, *P* = 0.548). All women with switches opted for long-acting implants or IUD from a less effective contraceptive method (condoms). However, fewer women (15, 9.6%) discontinued family planning for at least one week within the first year after birth for the intervention group compared to 37 (24.2%) women in the control group (OR = 0.34; 95% CI = 0.17-0.65, *P* = 0.001). The reasons for discontinuing family planning did not differ by group, except for method-related side effects which were less reported in the intervention group compared to the control group (OR = 0.25; 95% CI = 0.13-0.58, *P* = 0.001). The different sub-group analyses did not indicate any differences in family planning continuation between groups across any ART adherence level categories reported throughout the study period.

While we performed a randomized control trial and anticipated that any differences in baseline characteristics occurred by chance, we detected baseline differences in children living in a specific household, parity, prenatal visits, household income, domestic violence, religion, disclosure to spouse, and duration on ART (**Table 1**). We assessed whether the estimated odds ratio was affected by differences in baseline characteristics between groups by fitting multivariable logistic regression models. In these models, we found no meaningful change in the odds ratio of confirmed consistent/continuous use of an effective contraceptive method at 12 months for intervention vs control participants after adjustment for the factors listed above (adjusted OR = 2.15; 95% 95% CI = 1.35-4.41; *P* = 0.004). In stratified analyses to assess for differences in our primary outcome within sub-groups, no sub-group-by-treatment interaction terms were significant (**Table 3**). Thus, while we never set out to estimate effects within sub-groups, our results do not suggest differential effects in treatment within specific sub-groups of WLWH.

**Table 3:** Maternal baseline subcategories by study arm with confirmed continuous use of an effective contraceptive method at 12 months

Sub-group (n)	Routine care (n = 159)	FP voucher (n = 158)	Odds ratio (95% CI)	*P-*value	Adjusted odds ratio	*P*-value	*P*-value for interaction term
**Children <18 years:**							
<3	69/101 (68.3%)	67/81 (82.7%)	2.24 (1.12-4.97)	0.001	1.00 1.82 (1.12-3.26)	0.003	
≥3	41/58 (70.7%)	59/77 (76.6%)	1.32 (1.07-2.35)	0.067			0.981
**Age (years):**							
18-29	60/80 (75.0%)	69/72 (95.8%)	2.15 (1.42-5.19)	0.001	1.00		
≥30	50/79 (63.3%)	57/86 (66.3%)	1.27 (1.02-3.14)	0.132	1.75 (1.17-2.64)	0.003	0.097
**Parity:**							
<3	50/117 (52.1%)	76/99 (76.8%)	1.79 (1.36-3.01)	0.003	1.00		
≥3	60/42 (52.4%)	50/59 (84.7%)	1.73 (1.07-2.92)	0.002	1.68 (1.15-2.34)	0.002	0.797
**Prenatal visits:**							
<4	44/64 (68.8%)	34/40 (85.0%)	2.27 (1.67-6.51)	0.003	1.00		
≥4	66/95 (69.5%)	92/118 (78.0%)	1.35 (1.16-3.37)	0.031	1.87 (1.10-3.18)	0.014	0.280
**Household income (Ugandan shillings):**							
≤150 000	98/122 (80.3%)	98/105 (93.3%)	1.56 (1.32-2.13)	0.003	1.00		
>150 000	14/37 (37.8%)	28/53 (52.8%)	2.14 (1.48-7.12)	0.003	1.72 (1.32-3.63)	0.013	0.772
**Domestic violence:**							
No	96/141 (68.1%)	101/128 (78.9%)	1.97 (1.04-2.75)	0.001	1.00		
Yes	14/18 (77.8%)	25/30 (83.3%)	0.46 (0.23-3.59)	0.265	1.72 (1.26-2.14)	<0.001	0.623
**Disclosed to spouse:**							
No	11/23 (47.8%)	13/16 (81.3%)	2.14 (1.25-6.07)	0.003	1.00		
Yes	99/136 (72.8%)	115/142 (81.0%)	1.72 (0.87-3.22)	0.003	1.81 (1.25-3.63)	0.033	0.241
**ART duration (years):**							
<4	58/69 (84.1%)	60/63 (95.2%)	2.06 (1.36-7.15)	0.002	1.00		
≥4	52/90 (57.8%)	66/95 (69.5%)	1.59 (1.02-3.17)	0.003	1.98 (1.14-2.69)	0.002	0.572
Ever used modern contraception:							
No	28/52 (53.8%)	35/55 (63.6%)	1.67 (1.44-2.43)	0.143	1.00		
Yes	82/107 (76.6%)	91/103 (88.3%)	1.92 (1.23-4.22)	0.003	2.24 (1.47-5.92)	0.003	0.216

ART – antiretroviral therapy, FP – family planning

## DISCUSSION

We demonstrate that sustained and structured family planning support facilitates consistent use of effective postpartum contraception amongst WLWH in Southwestern Uganda. We found an 11% increase in consistent use of contraception among women enrolled in the family planning support group (80%) vs women in standard of care (control) group (69%). We also found a 7% lower rate of pregnancy in the intervention group (2%). Women who started early use of contraception within 3 months postpartum were more likely to maintain contraceptive use than those starting later than three months postpartum. There was a 10% rate reduction in the desire to have another pregnancy/child in two years postpartum among women in the intervention group (3%) vs control (13%). More women in the control group discontinued family planning mostly because of method-related side effects compared to intervention group. There were similar rates of women who had ever-enrolled on any family planning method within the course of the year in both groups, although active-use of contraception at 12-month postpartum reduced significantly in control vs intervention groups. While not statistically significant, we did observe a higher rate of change/switching contraceptive methods in the intervention vs control groups, with different methods used for those ever enrolled on an effective family planning method within the course of one year. Only proportions of women enrolled on implants were statistically significant. These data therefore demonstrate that, consistent or continuous use of contraception could be increased by a well-structured comprehensive family planning voucher program inclusive of structured and dedicated one-on-one counseling to: reassure, provide proper information, understand and address socio-cultural concerns, support and ensure correct and continuous use of contraception, as well as a good follow-up mechanism to improve provider-client-interaction and remind users of appointments for review or refill to avoid unintentional discontinuation due to missing clinically allowable grace period.

Our data contribute to existing evidence on optimal uptake and utilization of family planning to avert unwanted pregnancies, child mortality and maternal deaths when couples are well supported to adequately space pregnancies in resource-limited settings. Like other prior studies [39,40], we found a modest increase in continuous use of contraception among women supported to initiate and use different methods of contraception. Other scholars have also documented reduced unwanted pregnancy rates when women are well counseled and given adequate information to quickly initiate postpartum contraceptive use during antenatal or postnatal visits [41], plus other secondary outcomes, including reduced contraceptive method discontinuation [42], reduced pregnancy intentions [18], improved method switching/ change [43], uptake and long-term contraceptive use among postpartum women [4,18]. Our data therefore signal that, among WLWH qualified to use available postpartum contraception, sustained structured family planning support through structured one-on-one counseling, improved follow-up through additional health information, routine reviews and scheduled SMS reminders to prompt refills during this critical postpartum period using the existing structures of a publically funded hospital significantly improves uptake and adherence to effective contraceptive methods. This approach seems to facilitate proper information transfer, continuity, correct use and reassurance about anticipated side effects, thus providing efficient, convenient, low-cost means to reduce delayed refills, delayed switching and discontinuation duration in a resource-limited setting where uptake to freely available contraceptive methods are mainly constrained by method acceptability and information gap [6]. The significant difference in other clinically significant outcomes between groups, also offer promising preliminary data that structured family planning support is useful in delaying pregnancy intentions, as well as method discontinuation due to side effects in this select population that access regular and routine HIV care at designated specialized HIV clinics. Whether other populations gain preferential benefit from the structured family planning support over standard of care remains an important question for further investigation.

Our results are largely consistent with prior studies comparing the effect of improved quality of care through intensive counseling at initial visit/consultation and multiple contacts or technological reminders with routine care for reducing rates of unwanted pregnancies as well as improving continuation rates and acceptability to contraception. We acknowledge two 2013 (9 trials,) and 2019 (10 trials, n = 6242) systematic reviews reporting on strategies to improve adherence and continuation of hormonal methods of contraception [39,44]. However, only one prior trial (n = 43) has specifically compared a multi-component intervention of a one-on-one nurse counseling, a videotape about OCs and written material about OCs during antepartum period vs routine resident-physician counseling (usual care), exclusive of any technological reminders or calls [45]. Also noteworthy from both reviews, no adherence, consistent use data was reported among trials investigating use of direct in-person counseling or multiple components of counseling during initial visit or at discharge. Additionally, in both reviews, neither pregnancy nor discontinuation data were reported among trials investigating use of reminders and or educational messages compared to routine care.

As we acknowledge documented benefit of various strategies to improve contraceptive adherence and continuation, we note that several of these trials had small sample sizes, and only two involved technological approaches. The evidence in the recent review was also largely limited by variability of comparator “usual care” and heterogeneity of outcome definitions. For example, Trent and colleagues defined on-time injection appointments as adherence in an intervention that included daily appointment text messages 72 hours before a scheduled clinic visit, a call following missed appointment and monthly health messages, where standard of care included counseling, clinic appointment reminders as well as a call from the nurse manager whenever a scheduled re-injection appointment was missed [46]. Bereson and colleagues [47] on the other hand, defined and assessed consistent OC use (adherence) and pregnancy from both self-report and medical records audit after a 6-month intervention of counseling plus weekly, monthly and toll-free phone calls compared to standard care from a nurse practitioner with written protocols for new OC users. Two other trials assessed number of missed pills per cycles one through three [48] or at six months, use of OC at last sexual encounter and interruptions in OC use longer than seven days, [49]. Importantly, all the 10 trials included in both reviews had high lost-to-follow-ups of 24%-44%. Unlike these prior studies, our study was powered to demonstrate a significant benefit (or lack thereof) of a multi-component intervention of sustained family planning support (inclusive of structured one-on-one counseling at discharge, and a structured follow-up-mechanism through additional health information, reviews and SMS reminders during this critical postpartum period) on consistent use of postpartum contraception compared to standard of care amongst WLWH in Southwestern Uganda.

Importantly, although scholars have documented up to a third of women starting a modern contraceptive method discontinuing the method within the ﬁrst year [6], our trial’s discontinuation rate was 24% amongst women receiving standard of care vs 10% for the intervention group. Although the provision of a structured follow-up mechanism inclusive of SMS reminders and or reviews, as well as additional health information about family planning benefits and expected possible side-effects on the voucher could have been helpful in continuously supporting women on different contraceptive methods chosen, qualitative data analysis of interviews conducted with a subset of participants to understand how this intervention facilitated couples’ or individual decision-making to initiate and continue contraception is ongoing. Another potential explanation for differences between our study and prior data, which have shown larger contraception use rates is probably our inclusion of high risk WLWH already enrolled at specialized HIV/ART clinics where they receive routine medical reviews, counseling and ART refills. Our selection criteria could therefore over-estimate true differences in contraception uptake and or consistent use within the general population, and specifically in women at lower risk for unmet contraceptive need. Over 80% of referent pregnancies in our study population were also reported as planned.

Although our study documented increased rates of discontinuation due to side effects with control vs family planning support group as previously reported [44], women who started early use of contraception immediately postpartum had better continued use of contraception than those starting later than three months postpartum. Additionally, the benefit of a well-structured and sustained multi-component intervention for improving effective contraceptive use was seen across most sub-groups. Effect sizes appeared more in certain sub-groups, for example women aged ≥30 years of age, those with parity ≥3, attended ≥4 prenatal visits, women who earn/have an household income >150,000UGX (1US$ = 3650UGX), and women that had ever used modern family planning, which corroborates prior work demonstrating consistent less likelihood of discontinuing contraceptive use in these categories [6,50]. However, there were no significant differences in the effect of the confirmed consistent use of a contraceptive method across these categories.

Our study had a number of strengths. This study documents consistent or continuous use of contraception in a randomized controlled trial amongst postpartum WLWH followed up over a 12-month period. We also document unintended pregnancies, failure, method switch, pregnancy intentions as well as reasons for discontinuation. Our research assistants and data analysts were blinded to the group allocation and hypothesis of the study. A different RA from the one enrolling the participants collected follow-up data to limit social desirability bias. Our data are also the first to our knowledge powered to detect a difference in continuous contraceptive use with a sustained and structured multi-component family planning support compared to the standard of care. We also performed this randomized controlled trial in a regional referral, prototypical, publically-funded and operated hospital in a rural setting with an active postnatal and family planning unit, subject to standard limitations of public sector health care facilities in the region. As such, the study has great potential for generalizability to similar settings. Given that most women in our study (86%) had reportedly disclosed to at least one of their sexual partners, we observed a low rate of eligible participants declining participation (N = 44, 8%) mainly due to worry for possible unintended sero-status disclosure (N = 28, 64%) or limited time available to them to participate in the study (N = 16, 36%). The population of postpartum WLWH with a high proportion of contraception uptake also enabled us to document the differences in contraceptive uptake, continuation, switch and discontinuation of the initiated contraceptive method and the effect of baseline subcategories by study arm with consistent use of contraception within a multivariate model.

Our study had some important limitations. We observed an increase in contraceptive uptake from 38% to 98% and pregnancy intentions dropped from 55% to 8% from the pre-study period to the study period, suggesting either presence of strict inclusion criteria of only WLWH, all already enrolled and receiving ART from designated HIV clinics. This may also suggest a possibility of a Hawthorne effect, which might have resulted from improved availability of contraception different method choices at the facility within easy reach of all women regardless of the group or involvement in the study, as well as improved provider-client-interaction from the well-trained facility nurses that administered these methods and provided good, one-on-one counseling throughout the study period as recommended by the standard of care guidelines vs routine care that is frequently practiced in similar publically-funded facilities. As such, similar rates of women who had ever enrolled on any family planning method within the course of the year in both groups were observed. We observed a striking percentage of more than 80% intended pregnancies in this study. We hypothesize that the reason for this high rate of intended pregnancy is possibly due to the ongoing Test and Treat policy in Uganda, enrolment of all our participants on ART (with mean ART duration of 5.1 (SD = 4.5) for those in the voucher group and 4.1 years (SD = 3.3). This prior enrolment on ART may have improved counseling and knowledge for improved behavior change. Less than 40% of women also reported use of modern contraception 2 years’ pre-pregnancy, probably in anticipation of pregnancy. We further note a small proportion of women who desired pregnancy in the first year following delivery (4 vs 5 women) and a small rate of pregnancies (3 vs 14 pregnancies) that made it difficult to make meaningful comparisons between intended and unintended pregnancies.

Another limitation of our study was the inability to singly identify an aspect of this particular multi-component intervention that influenced consistent use of contraception. Although our study was matched to include male partners, few males participated. However, the measurement of our study outcomes did not require partner participation, suggesting generalizability and replicability of our study findings.

## CONCLUSION AND RECOMMENDATIONS

We found that sustained and structured family planning support inclusive of structured one-on-one counseling at discharge and an improved follow-up mechanism facilitates consistent use of effective contraception amongst postpartum WLWH in rural southwestern Uganda. Pregnancy rates were therefore rare in the family planning support group. Women who were initiated to start use of contraception within 3 months following birth had better continued use of contraception than those starting later than three months postpartum. There were similar rates of changes/switches in postpartum contraception use with different methods for those ever enrolled on an effective family planning method within the course of one year. There was also a significant reduction in the desire to have another pregnancy/child in two years postpartum among women in the intervention group vs control. Active enrolment on any family planning method at 12 months postpartum was better in the family planning support group. Given the increased use of modern contraception, especially injectables in this population, there is clear need to improve family planning support from the context-aware health care providers through a structured one-on-one counseling before discharge, as well as active follow-up to facilitate provider-client interaction, early initiation, continuous use, and adequate switching to reduce unnecessary/unintended discontinuation and unplanned pregnancies. This is particularly important in resource-limited settings where women’s family planning needs differ over time from their sexual partners and their negotiation skills are lacking, especially amongst women having irregular sexual partners/encounters due to separation, migration, work, social lifestyle or spousal rejection of modern contraception use. Improved quality of care at consultation and or initial visit, support and provision of additional health information could also improve continuation rates, reduce unwanted pregnancies and facilitate adequate referral or switching to a more tolerable effective contraception option.

This work documents postpartum contraceptive use dynamics among WLWH through a client-specific prospective trial over the 12month period. The trial reflects the effectiveness of an intervention aimed at reducing unwanted/unplanned pregnancies, through improved information transfer using one-on-one face-to-face counseling, availing educational reference materials, and an appropriate follow-up mechanism to continuously engage and encourage family planning initiation and consistent use. There is therefore a need to scale up this model in all maternity centers to help curb unplanned pregnancies, maternal morbidity and mortality. Further work should help clarify which methods are related with which side effects or health concerns so as to help reduce the likelihood of these negative expressions. Additionally, further evaluation of the actual and perceived barriers to continuous use of modern contraception in such resource-limited settings will help improve its availability and consistent use in such settings.
